# A subset of epithelial cells mimics regulatory T cells and contributes to immune evasion during development of pancreatic adenocarcinoma

**DOI:** 10.1186/s12916-020-01620-y

**Published:** 2020-06-29

**Authors:** Courtney W. Houchen, Min Li

**Affiliations:** Department of Medicine, The University of Oklahoma Health Sciences Center, 975 NE 10th Street, BRC 1262A, Oklahoma City, OK 73104 USA

**Keywords:** Pancreatic cancer, Epithelial cells, Regulatory T cells, Immune evasion

## Abstract

Pancreatic cancer is refractory to most current treatment options. Immunotherapy emerges as an effective and novel therapeutic strategy for several solid tumors. However, most of the clinical trials on immunotherapy have failed in pancreatic cancer. Understanding the underlying mechanism that drives immune evasion of pancreatic cancer is critical for overcoming resistance to therapy. Recently, Dr. He Ren and colleagues proposed a novel concept that a subset of epithelial cells in pancreatic cancer mimics the phenotype and function of regulatory T cells, named as “quasi-regulatory T cells.” These cells contribute to enhanced immune evasion, angiogenesis, and metastasis of pancreatic cancer, thus providing potential therapeutic targets to improve the sensitivity of immunotherapy for this devastating disease. This ground-breaking concept will advance our understanding on the immune evasion of pancreatic cancer and chart novel paths towards the development of personalized treatment for pancreatic cancer.

## Background

Pancreatic adenocarcinoma (PDAC) remains the most lethal cancer with an overall 5-year survival rate of 10%. Current standard therapies for PDAC have rarely achieved long-term remission even in patients at early stages. Moreover, PDAC appears to be refractory to the immunotherapy, partially due to the tumor immunosuppressive microenvironment (TIM) [1]. The recruitment of immunosuppressive cells such as MDSCs and Treg cells is a canonical mechanism that contributes to the formation of TIM. However, little attention has been paid to the role of intrinsic factors derived from malignant epithelial cells in driving immune evasion.

## Epithelial cells mimic regulatory T cells in PDAC

Dr. He Ren and colleagues have recently published a series of papers focusing on understanding the role of immune regulatory factors derived from malignant epithelial cells [2–7]. They found that FOXP3, a master transcription factor of Tregs, is also expressed in pancreatic cancer cells (named as cancer-FOXP3, or c-FOXP3) and transactivated CCL-5 to recruit FOXP3^+^Tregs to mediate immune evasion. This study uncovered the interaction between different types of FOXP3^+^ cells and indicated that the tumor-intrinsic factors could serve as potential biomarkers to predict the response of treatments that target Tregs [2]. Immune-checkpoint blockade (ICB) represents a novel treatment strategy in several solid tumors. Positive expression of PD-L1 in tumor tissue is recognized as an indicator of a better response rate to ICB treatment. But the underlying mechanism of PD-L1 upregulation in PDAC remains elusive, until very recently, Ren et al. reported that c-FOXP3 activated PD-L1 transcription and directly inhibited the activity of CD8^+^ T cells. Thus, c-FOXP3 represents a key factor in reprogramming the tumor immune microenvironment [3]. To date, several clinical trials evaluating monoclonal antibodies and inhibitors that target PD-1/PD-L1 or CCL-5/CCR5 pathway have been launched. The present study provides a rationale for the combination of PD-L1 and CCL5 inhibition to improve the response to immunotherapy in PDAC, especially in patients with high c-FOXP3 levels.

## Immunosuppressive cytokines contribute to angiogenesis and metastasis in PDAC

Interleukin 35 (IL-35) is a potent immune inhibitory cytokine primarily expressed by Tregs. Ren and collaborators found that IL-35 was highly expressed in the malignant epithelial cells of PDAC and associated with poor prognosis. Meanwhile, IL-35 stimulated ICAM1 expression via STAT-1 and mediated PDAC extravasation and metastasis [4]. This finding provides evidence that blockade of IL-35/ICAM-1 signaling pathway might be an effective strategy for the treatment of metastatic PDAC. More importantly, Ren recently provided convincing evidence that IL-35 stimulated angiogenesis of PDAC by recruiting monocytes into the malignant pancreatic tissues via CCL-5 and polarized the monocytes towards a pro-angiogenic phenotype to secrete CXCL1/CXCL8 [5]. CXCL1 and CXCL8 act through CXCR1/CXCR2 to activate MAPK signaling pathways and promote angiogenesis [8]. The present anti-angiogenetic reagents mainly target the VEGF/VEGFR pathway but showed disappointing results in several clinical trials [9]. Ren’s research elucidated a non-canonical, VEGF-independent angiogenesis that exists in PDAC, and anti-IL-35 antibody is an effective reagent to inhibit angiogenesis of PDAC. These results extend our understanding on the role of immunosuppressive cytokines to the pathogenic angiogenesis and metastasis of PDAC, thus presenting a novel target for the treatment of this devastating disease.

## EHF deficiency in malignant epithelial cells reprograms the metastatic phenotype and mimics the Tregs-like immunesuppressive function in PDAC

TGF-β is a classic immune inhibitory cytokine, expressed by multiple cell types including pancreatic cancer cells. Ren has recently found that EHF, one of the epithelium-specific ETS (ESE) transcription factors, negatively regulated the transcription of TGF-β [6]. In pancreatic tumorigenesis, pancreatic epithelial cells lost the expression of EHF via methylation and therefore secrete high levels of TGF-β to induce the conversion of Tregs. They further showed that EFH deficiency released the secretion of GM-CSF to promote MDSC accumulation and formed TIM. Meanwhile, loss of EHF promoted epithelial-mesenchymal transition (EMT) and metastasis of PDAC via the downregulation of E-Cadherin [7]. Tregs are developed from mesoderm and do not express epithelial transcription factor such as EHF. Therefore, deficiency of EHF in malignant epithelial cells would reprogram the phenotype and mimics the Tregs-like immunosuppressive function in PDAC.

## Conclusions

**Fig. 1:**
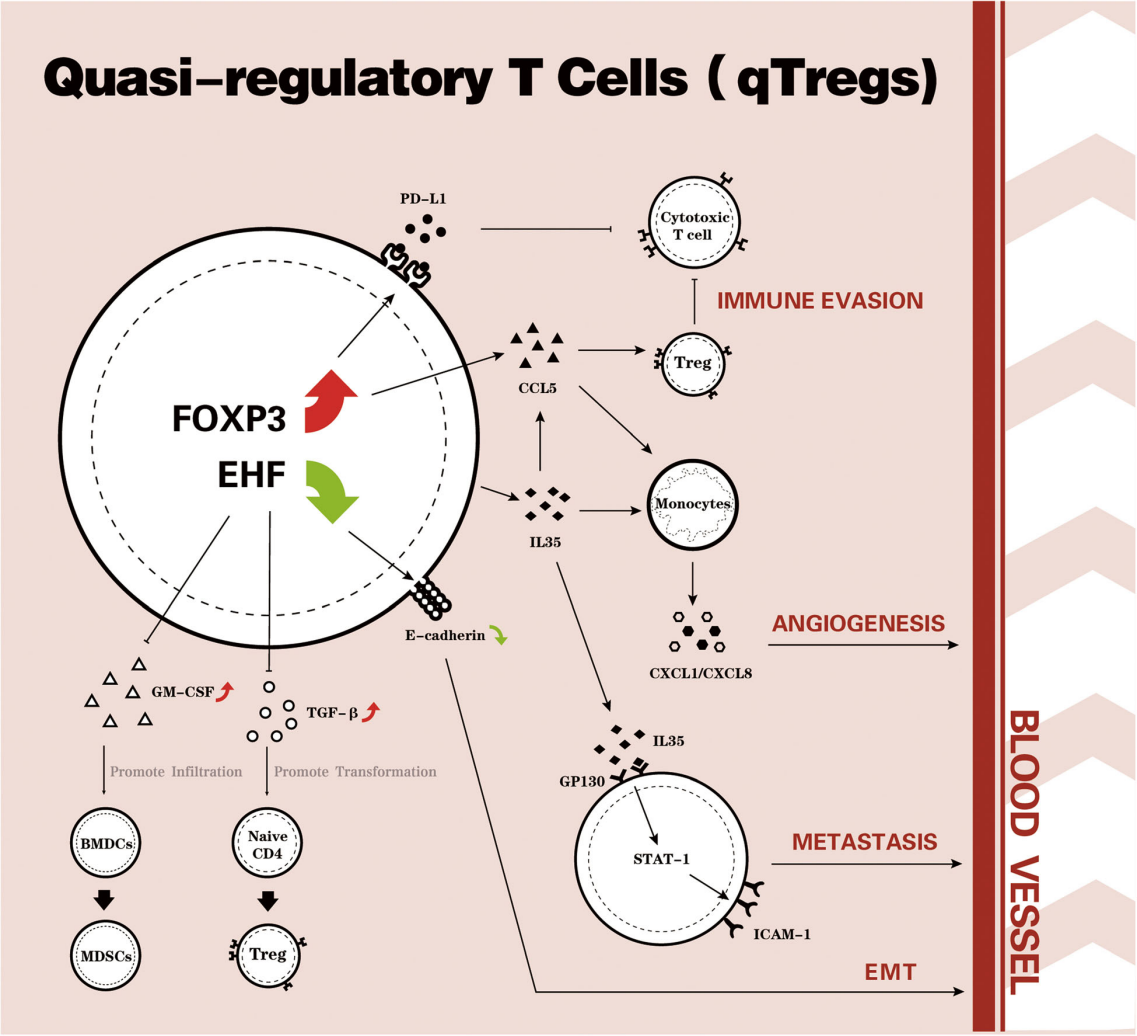
Schematic diagram of the phenotype and function of quasi-regulatory T cells in pancreatic adenocarcinoma. The quasi-regulatory T cells exist during pancreatic tumorigenesis through the upregulation of FOXP3 and downregulation of EHF, with specific surface markers (PD-L1 high and E-Cadherin low) and secreted cytokine profiles (TGF-β, IL-35, GM-CSF). Those cells play important roles in immune evasion, angiogenesis, and metastasis of pancreatic adenocarcinoma

Taken together, Ren and colleagues described the characteristics of cell-intrinsic factors that mimic the phenotype and function of Tregs. They firstly showed that a subtype of “quasi-regulatory T cells” exist during pancreatic tumorigenesis. These cells increased the secretion of several critical immune regulatory factors by the upregulation of FOXP3 and downregulation of EHF. In addition, overexpression of PD-L1 and loss of E-Cadherin could be used as the surface marker of “quasi-regulatory T cells.” Previous studies of TIM mainly focused on the infiltrating immune cells and stromal cells. This ground-breaking concept will advance our understanding on the immune evasion of pancreatic cancer and chart novel paths towards the development of personalized treatment for pancreatic cancer (Fig. 1). For clinical practice, the best strategy to personalize the immunotherapy is to find effective biomarkers to predict the sensitivity to the ICB treatment. Considering the complexity of the tumor microenvironment of PDAC, a recent study has recommended examining bulk tissue samples to predict the response of PDAC to immunotherapy [10]. However, 85% of PDAC are unresectable at first diagnosis, which means only biopsy specimens are available for these patients. Thus, “quasi-regulatory T cells” could serve as a better biomarker to select patients for immunotherapy.

## Future novel perspectives

In future studies, several key questions need to be answered to better understand the underlying mechanisms of “quasi-regulatory T cells” driven immune evasion of PDAC. First of all, during the development of pancreatic cancer, what is the master regulator that controls the formation of “quasi-regulatory T cells”? Secondly, how to identify the biological effect of “quasi-regulatory T cells” during pancreatic tumorigenesis? Last but not the least, what is the difference between the transcriptomic networks of quasi-regulatory T cells and regulatory T cells in the same PDAC tissue? Through further investigation of those questions, we should be able to better understand the biology of PDAC, especially on immune evasion, and develop novel therapeutic targets to improve the effectiveness of immunotherapy in PDAC.

## Data Availability

Not applicable
